# Inflammatory process in Alzheimer's Disease

**DOI:** 10.3389/fnint.2013.00059

**Published:** 2013-08-13

**Authors:** Marco A. Meraz-Ríos, Danira Toral-Rios, Diana Franco-Bocanegra, Juana Villeda-Hernández, Victoria Campos-Peña

**Affiliations:** ^1^Departamento de Biomedicina Molecular, Centro de Investigación y de Estudios AvanzadosMexico City, Mexico; ^2^Departamento de Fisiología Biofísica y Neurociencias, Centro de Investigación y de Estudios AvanzadosMexico City, Mexico; ^3^Posgrado en Ciencias Biológicas, Universidad Nacional Autónoma de MéxicoMexico City, Mexico; ^4^Departamento de Neuropatología Experimental, Instituto Nacional de Neurología y NeurocirugíaMexico City, Mexico; ^5^Laboratorio Experimental de Enfermedades Neurodegenerativas, Instituto Nacional de Neurología y Neurocirugía Manuel Velasco SuárezMexico City, Mexico

**Keywords:** Alzheimer disease, amyloid-β, neurodegeneration, microglia, astrocyte, neuroinflammation, pro-inflammatory cytokine, anti-inflammatory strategies

## Abstract

Alzheimer Disease (AD) is a neurodegenerative disorder and the most common form of dementia. Histopathologically is characterized by the presence of two major hallmarks, the intracellular neurofibrillary tangles (NFTs) and extracellular neuritic plaques (NPs) surrounded by activated astrocytes and microglia. NFTs consist of paired helical filaments of truncated tau protein that is abnormally hyperphosphorylated. The main component in the NP is the amyloid-β peptide (Aβ), a small fragment of 40–42 amino acids with a molecular weight of 4 kD. It has been proposed that the amyloid aggregates and microglia activation are able to favor the neurodegenerative process observed in AD patients. However, the role of inflammation in AD is controversial, because in early stages the inflammation could have a beneficial role in the pathology, since it has been thought that the microglia and astrocytes activated could be involved in Aβ clearance. Nevertheless the chronic activation of the microglia has been related with an increase of Aβ and possibly with tau phosphorylation. Studies in AD brains have shown an upregulation of complement molecules, pro-inflammatory cytokines, acute phase reactants and other inflammatory mediators that could contribute with the neurodegenerative process. Clinical trials and animal models with non-steroidal anti-inflammatory drugs (NSAIDs) indicate that these drugs may decrease the risk of developing AD and apparently reduce Aβ deposition. Finally, further studies are needed to determine whether treatment with anti-inflammatory strategies, may decrease the neurodegenerative process that affects these patients.

## Introduction

Alzheimer's disease (AD) is the most common cause of dementia in elderly adults. AD is clinically characterized by progressive loss of memory and other cognitive functions. Histopathologically is recognized by the presence of neuritic plaques (NPs) and neurofibrillary tangles (NFTs). The origin causes of AD can be classified as familial or sporadic. The incidence of familial cases is low (5–10%) and is related to the presence of mutations in three different genes: presenilin-1 (PS1), presenilin-2 (PS2), and amyloid-β precursor protein (APP-β) (Chartier-Harlin et al., 1991; Goate et al., 1991; Murrell et al., 1991; Levy-Lahad et al., 1995; Duff et al., 1996; Sisodia et al., 1999). Sporadic AD represents 90–95% of total cases, and although its etiology is multi-factorial, age is the main risk factor. Although there are different genetic and environmental causes, all patients have a similar clinical behavior and develop identical brain lesions: NFTs consisting of Tau (τ) protein and NPs consisting of amyloid-β (Aβ) peptides. These alterations are the final result of post-translational modifications and involve different genes and render AD as a complex multigenic neurodegenerative disorder.

In addition to this multi-genic complexity in AD, now we know that Aβ promotes an inflammatory response mediated by microglia and astrocytes, thus activating signaling pathways that could lead to neurodegeneration. It is currently unknown whether brain inflammation in AD patients is the cause of the disease or a secondary phenomenon. Although it was previously thought that the central nervous system (CNS) was an immune-privileged site, now is well known that certain features of inflammatory processes occur normally in response to an injury, infection or disease. The resident CNS cells generate inflammatory mediators, such as pro-inflammatory cytokines, prostaglandins (PGs), free radicals, complement factors, and simultaneously induce the production of adhesion molecules and chemokines, which could recruit peripheral immune cells. This review describes the cellular and molecular mediators involved in the inflammatory process associated with AD and several possible therapeutic approaches describe recently.

## NPs and Aβ in the neuro-inflammatory process

NPs, are extracellular deposits structures that contain a highly insoluble fibrillar Aβ core formed by fragments of 39–42 amino acids surrounded by microglia, reactive astrocytes, and dystrophic neurites produced from degenerating neuronal processes (Iversen et al., 1995). Aβ normally originates from APP-β (Kang et al., 1987) through the sequential action of beta secretase and the multi-protein gamma-secretase complex.

**Figure 1:**
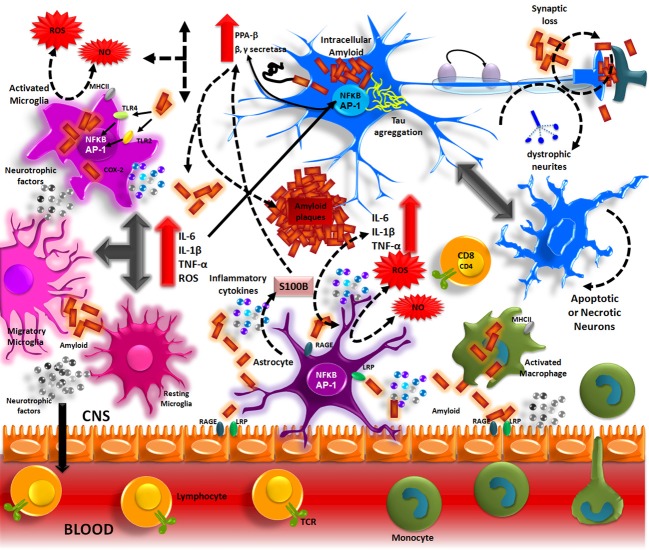
**Inflammation in Alzheimer's disease**. The Aβ peptide produced by APP processing, form aggregates that activate microglia through TLRs and RAGE receptors. These receptors in turn, activate NF-κ B and AP-1 transcription factors, which induce the reactive oxygen species (ROS) production and the expression of inflammatory cytokines (IL-1, IL-6, TNF). These inflammatory factors directly acting on the neurons and also stimulate the astrocytes, which amplify the pro-inflammatory signals, inducing a neurotoxic effects. The inflammatory mediators generate by resident CNS cells, induce the production of adhesion molecules and chemokines, which recruit peripheral immune cells.

Aβ accumulation in the brain, in one of the main pathological processes of AD patients. The formation of these deposits initiates a series of cellular events which are able to elicit an immune response where resident cells such as microglia and astrocytes could participate. Aβ accumulation in parenchyma and blood vessels causes microglial migration and promotes acute and chronic inflammatory responses against the aggregates, thus inducing the production of nitric oxide (NO), reactive oxygen species (ROS), pro-inflammatory cytokines (TNFα, IL-1β and IL-6), and PGs (PGE2), which eventually could promote neuronal death (Figure 1) (Akiyama et al., 2000; Kitazawa et al., 2004).

## Cellular mediators

### Microglia

Microglia are resident brain cells, derived from monocyte precursors cells during embryogenesis, and are able to provide the initial response against any injury that occurs in the CNS. Although these cell were observed by Nissl over 100 years ago, their definitive identification and characterization was performed by Mrak (2012). Activated microglial association around NPs suggested that microglia participate in the accumulation of the Aβ observed in AD patients (Glenner et al., 1984); this hypothesis was supported by several studies (Rozemuller et al., 1986; Dickson et al., 1988).

Normally, microglia exists in an inactive state; morphologically, these cells have a small soma with branching processes. When activated by pathogens or injury, these cells suffer visible morphological changes, including decreased branching and soma growth, the acquisition of an amoeboid form and display a wide variety of specific cellular surface markers (Town et al., 2005). NPs in AD patients' brain activate the inflammatory response mediated by microglia and cause pro-inflammatory cytokine secretion, which may directly cause neuronal injury.

Microglia *in vitro* cell cultures are able to phagocyte the amyloid peptide. However, ultrastructural analysis of tissues from AD patients demonstrated that there is no presence of amyloid fibrils in the lysosomal compartments of local microglia cells (Frackowiak et al., 1992). Whereas microglia has the ability to phagocyte Aβ *in vitro*, its phagocytosis capacity is limited. An important observed feature was the abnormal presence of macrophages infiltrating from the periphery, which showed amyloid fibers in their lysosomal compartments (Wisniewski et al., 1991; Akiyama et al., 1996). Currently, we know that there are two types of phagocytic cells within the CNS that are able to initiate the innate immune response: microglia and peripheral macrophages (Rezai-Zadeh et al., 2009; Gate et al., 2010). These macrophages are recruited into the CNS by specific cytokines and chemokines, which are released during microglial and astrocytic activation and are able to cross the blood-brain barrier.

Similarly to macrophages, microglia recognizes pathogens through pattern recognition receptors (PRRs), which include specific toll-like receptors (TLRs), nucleotide-oligomerization binding domain (NOD) proteins, and C-type lectin receptors. These receptors interact with pathogen-associated molecular patterns (PAMPs) or damage-associated molecular patterns (DAMPs) to initiate the cellular defense mechanisms (Sterka and Marriott, 2006; Rubartelli and Lotze, 2007). Thus, the formation and release of ROS, pro-inflammatory cytokines (IL-1β, IL-6, TNF-α, and IFN-γ) (Lue et al., 2001), chemokines (MIP1α, MIP1β, RANTES, and MCP1), and growth factors such as macrophage colony-stimulating factor (MCSF) and complement factors (C1q, C3, C4, and C9) (Walker et al., 1995) begin. In addition to the before mentioned molecules, microglia is also able to express receptors for advanced glycosilation end products (RAGE), Fc receptors, CD40, FP receptors, and various scavenger-type receptors (El Khoury et al., 1998; Tan et al., 1999; Walker and Lue, 2005; Okun et al., 2010). Although microglia has the capacity to respond to various stimuli, the presence of Aβ is particularly important. Aβ induces a high accumulation of surface molecules of type I and II major histocompatibility complex (MHC) (McGeer et al., 1988).

Microglial cells primarily have an immunomodulatory role and express a large variety of immune response-related antigens and molecules; however, the specific role of microglia within the CNS remains controversial. Microglial activation is not a simple and unique phenomenon; instead, activation is a continuous series of events in which microglia awakens innate and adaptive phagocytic immune responses and consistently display activation through antigens on the cellular surface (Town et al., 2005). Under this principle, microglia displays a “variable” response, in which a mixture of the classical activation pathway and exacerbated increase of alternative activation can be observed in AD patients, which could leads to irreparable damage that result in persistent neurodegeneration.

### Astrocytes

Astrocytes are the most abundant glial cells present in the CNS and have important functions in brain organization and maintenance (Sofroniew and Vinters, 2010). These cells interact with neurons and are involved in neurotransmitter secretion and recycling, ion homeostasis, energy metabolism regulation, synaptic remodeling and modulation of oxidative stress, information processing, and signal transmission (Halassa and Haydon, 2010; Henneberger et al., 2010).

In early stages of AD, activated astrocytes are located in two regions: the molecular layer of the cerebral cortex and near the amyloid deposits below the pyramidal cell layer. The mechanisms leading to the activation of these cells in response to the pathological changes produced by AD are not obvious, but it has been demonstrated that the presence of amyloid activates astrocytes. Activated astrocytes can phagocytose and degrade amyloid, which suggests that they importantly contribute to the removal of accumulated Aβ in parenchyma. Astrocytes and microglia are activated through TLRs and RAGE-dependent pathways, thus causing local inflammation that eventually could intensifies neuronal death.

Overall, the inflammatory process in AD patients is shown by changes in microglial morphology and astrogliosis, which is manifested by an increase in number, size and motility of astrocytes. Astrocyte activation is present in many neurodegenerative conditions, expressing high levels of glial fibrillary acidic protein (GFAP), vimentin, and nestin. Unfortunately, these changes cause a “disruption” of normal activities in astrocytes, which are essential for normal neuronal function. Maintenance of glutamate concentration in the extracellular space is among the normal physiological functions; altering homeostasis causes a local neuron depolarization, which leads to citotoxic damage. Thus, although astrocytic activation has a protective role in brain, intense activation exacerbates neuronal damage and accelerates disease progression.

Similarly to microglia, astrocytes quickly respond to injury; these cells are located near the fibrilar Aβ deposits, which are responsible for the astroglial activation observed in AD patients. Injection of Aβ oligomeric forms into the retrosplenial cortex of rats caused a significant astrocyte activation, as shown by transcription factor NF-κ B activation and the presence of inflammatory mediators such as tumor necrosis factor (TNF-α), interleukin 1 (IL-1β), and cyclooxygenase-2 (COX-2)(Carrero et al., 2012). The activated astrocytes express on their cell surface, receptors that bind the Aβ peptides such as RAGE, receptor-like density lipoproteins, proteoglycans and various scavenger receptors (Wyss-Coray et al., 2003; Medeiros and Laferla, 2013). Thus, activated astrocytes are able to cause neurodegeneration and express inflammation-associated factors, such as S100β, consequently inducing neurite outgrowth. S100β expression correlates with the number of dystrophic neurites in AD patients (Mrak et al., 1996).

In astrocytes, NF-κB significantly controls chemokine and adhesion molecules secretion, favors peripheral lymphocyte infiltration and increases inflammatory response (Moynagh, 2005); this process becomes a self-regulating mechanism, which leads to neurodegeneration.

### Oligodendrocytes

Although oligodendroglia is important to maintain neuron morphology and function, little is known about how they are affected by Aβ deposits However, there are studies that indicate changes in white matter and abnormalities in myelin in AD patients (Kobayashi et al., 2002; Roth et al., 2005). Specifically, aberrations have been reported in the white matter of asymptomatic familial AD patients, particularly in patients with mutations in PS1 (Ringman et al., 2007). The presence of Aβ in oligodendrocyte cultures caused cell death. Although cell death can be prevented by the presence of anti-inflammatory agents, morphological alterations of the cells persist, suggesting that the damage cannot be completely reversed (Roth et al., 2005). Subsequently, oligodendrocyte differentiation and function is affected by the simultaneous presence of mutations in PS1 (hPS1^*M*146*V*^) and Aβ accumulation, as demonstrated in a mouse model. These abnormalities can lead to the development of abnormal patterns of myelin basic protein (MBP) (Desai et al., 2011), which completely alters oligodendrocyte homeostasis. Finally, the loss of trophic support provided by these cells might lead to increased neuronal vulnerability and inflammation, thus favoring neurodegeneration.

## Molecular mediators of inflammation in AD

**Figure 2:**
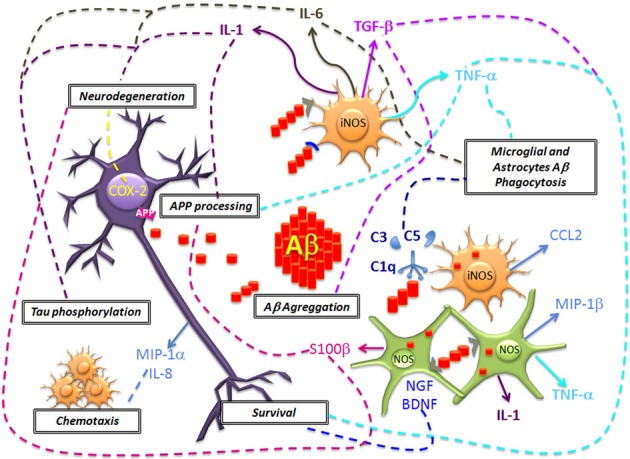
**Neuronal damage and Aβ deposition triggers microglial and astrocytes activation and the generation of inflammation molecular mediators**. The acute production of molecules of the complement system (C1q, C3, and C5), pro-inflammatory cytokines (IL-1, IL-6, TNF-α), chemokines (CCL2, MIP-1α, MIP-1β, and IL-8) mediate the Aβ clearance. However, in a chronic stage these molecules could promote an increased expression and alteration of APP processing, Aβ deposition, Tau phosphorylation and neurodegeneration. Also, another effect of glial cells includes the generation of NO that promotes oxidative stress. The inflammatory microenvironment favors the production of COX-2 in neurons that leads to apoptosis. In contrast, it has been proposed that glial cells could mediate neuronal survival, by the production of TGF-β and neurotrophic factors (BDNF and NGF), but the disease progression results in failure to repair neurons.

The Aβ deposition activates the acute immune response of microglial cells and astrocytes. At the same time, amyloid plaques are responsible for the production and the activation of inflammation-related proteins such as complement factors, acute-phase proteins, chemokines and cytokines like interleukin 1 (IL-1), interleukin 6 (IL-6), tumor necrosis factor α (TNF-α), and transforming growth factor β (TGF-β). These molecular mediators of inflammation have been linked with a series of concomitant deleterious and beneficial effects (Figure 2).

### The complement system

The complement system is an important innate and adaptive immune response effector. This system is composed of a number of proteins and proteases that are activated in cascade (Forneris et al., 2012), and it appears to have a fundamental role in neurodegenerative diseases. Overall, the complement system uses the C1q molecule, mannose-binding protein or the interaction with the C3 multifunctional protein to recognize certain molecular patterns on pathogens. C3 activation recruits cells with phagocytic activity, and Membrane Attack Complex (MAC) is formed by binding C5–C9 (Ricklin et al., 2010). In adaptive immune response, the complement system is involved in T-cell response regulation and T-helper lymphocyte differentiation (Pekkarinen et al., 2011).

In neurodegenerative diseases, there is a deregulation of the classical complement pathway. Studies in AD patient brains have revealed an increase in the immunoreactivity of C1q, C3b, C4d, C5b-9, and MAC surrounding senile plaques (McGeer et al., 1989; Rogers et al., 1992) and microglia surrounding the fibrillar aggregates of Aβ in the microvasculature (Fan et al., 2007). The co-existence of these molecules with the Aβ aggregates suggests a possible link between classical complement pathway activation, inflammation and pathological aggregation of Aβ. A decrease in C1q levels has been reported in cerebrospinal fluid (CSF) of AD patient and this result contrasts with an increase of C1q levels in the CNS (Smyth et al., 1994). *In vitro* studies revealed that C1q recognizes fibrillar and aggregated forms of Aβ 1–42 and Aβ 1–40, but not the monomeric forms. The C1q receptor is expressed in microglia, which contributed to the belief that the increase of this molecule in AD patient brains could affect the phagocytosis of Aβ. However, in primary microglia rat culture studies, exposure to Aβ 1–42 and a nanomolar concentration of C1q caused a decrease in the phagocytosis of Aβ, compared to cultures exposed only to Aβ 1–42 (Webster et al., 2000). A similar result was observed in a transgenic mouse model, which expressed mutated hAPP and an absence of C1q expression (APPQ^−/−^). These mice showed no change in the amount of Aβ aggregates, but a decrease in glial cells activation was observed (Fonseca et al., 2004), thus establishing that C1q importantly contributes to microglial activation.

Microglia cells also produce C3. When these cells are exposed to Aβ synthetic peptides, their activation can be observed as well as an increase in C3 synthesis to 5-to 10 fold (Haga et al., 1993). When this molecule is inhibited, in the brain of an hAPP transgenic model, by the overexpression of the soluble form of the protein related to the complement receptor (sCrry), a 2- to 3-fold increase in Aβ deposits formation was observed in one-year-old hAPP/sCrry mice, which was accompanied by extensive neurodegeneration (Wyss-Coray et al., 2002). APP/C3^−/−^ double transgenic generation confirmed that the molecule modulated the microglia phenotype, and promotes Aβ degradation (Maier et al., 2008).

Complement receptor 1 (CR1) is expressed in cells with phagocytic activity, C3b and C4b are CR1 ligands, and their binding facilitates phagocytosis. Genome-wide association studies (GWAS) in AD patients have shown a direct relationship between CR1 gene variants with cognitive impairment in patients and an increase in amyloid plaque formation (Chibnik et al., 2011).

The anaphylatoxin (C5) has been linked to excitotoxicity and apoptosis activation in the CNS, which is believed to play an important role in the development of neurodegenerative diseases. This molecule also promotes chemotaxis and glial cells activation. In AD animal models, the use of the C5a receptor antagonist (C5aR or CD88) reduces the amount of Aβ aggregates and hyperphosphorylated tau protein (Fonseca et al., 2009; Ager et al., 2010). It appears that the complement system activation in AD might have beneficial effects for Aβ clearance; however, this activation could eventually become deregulated and favor neurotoxic effects by promoting unwanted inflammation. For these reasons, more studies are required to understand the changes affecting the complement system in AD development.

### Cytokines

Immune system cells are able to produce cytokines, which are soluble proteins that mediate inflammation. In the CNS, cytokines are produced by microglial cells and astrocytes and play a key role in CNS development during the embryonic stages. Cytokines are involved in the inflammatory processes of neurodegenerative diseases. In AD, these proteins may be important in the development of the pathology. Studies of AD patient tissues and CSF have shown elevated levels of pro-inflammatory cytokines such as IL-1β, IL-6, IL-10, TNF-α, and TGF-β (Blum-Degen et al., 1995; Tarkowski et al., 2002; Mrak and Griffin, 2005; Jiang et al., 2011). The increase of these cytokines is strongly related to microglia activation by exposure to Aβ aggregates (Meda et al., 1999). Animal models that overexpress the mutant hAPP protein have shown a direct relationship between the amount of Aβ aggregates and elevated levels of TNF-α, IL-6, IL-12, IL-1β, and IL-1α (Patel et al., 2005). In particular, studies have been performed seeking the relationship between each cytokine with AD.

### IL-1

IL-1 is known to be able to induce APP-β mRNA expression in endothelial cells (Goldgaber et al., 1989), which suggests that IL-1 increasing in AD patients could be linked to Aβ formation. In a study with AD patients, it was also proposed that IL-1 was produced by microglial cells surrounding NPs and this cytokine could promote S100β synthesis in astrocytes (Griffin et al., 1989). Subsequently, it was discovered that IL-1 was a contributing factor to initiate dystrophic neurite formation in Aβ diffuse deposits (Griffin et al., 1995). Similarly, the exposure of primary cultures of rat cortical neurons to S100β promotes dystrophic neurite formation and APP-β mRNA expression (Li et al., 1998). Thus, IL-1 might promote the Aβ formation and neuronal degeneration through S100β present in reactive astrocytes. Additionally, the increase in IL-1 in AD patients might promote an increase in p38-MAP kinase activity, which could lead to Tau hyperphosphorilation (Sheng et al., 2000; Li et al., 2003). IL-1β signaling blockade decreases GSK-3β activity and Tau phosphorylation and promotes neurogenesis through the Wnt/β-catenin pathway (Kitazawa et al., 2011). However, it has been recently suggested that IL-1β could promote Aβ removal (Matousek et al., 2012).

### IL-6

IL-6 is produced by microglial cells and is involved in the immunoreactivity present in patient tissues with clinical dementia (Hull et al., 1996). Similarly to IL-1, IL-6 promotes APP-β expression (Ringheim et al., 1998) and could also contribute to NFT formation by inducing Tau phosphorylation through cdk5/p35 pathway deregulation (Quintanilla et al., 2004). However, IL-6 overexpression has been observed in the brain of two hAPP transgenic models (TgCRND8 and Tg2576). This overexpression resulted in significant gliosis, a decrease in Aβ deposits in TgCRND8 mice brains due to phagocytic marker upregulation in glial cells and increasing microglial Aβ phagocytosis. IL-6-induced neuroinflammation, and has not any effect on APP-β processing in TgCRND8 mice, which suggests that reactive gliosis, could have a beneficial effect in early stages of disease by promoting Aβ elimination (Chakrabarty et al., 2010).

### TNF-α

TNF-α is a cytokine that can have beneficial or harmful effects on different neurons. This cytokine stimulates NF-κB transcription factor which, induces the pro-inflammatory molecules expression, and promote the synthesis of neuronal survival factors such as calbindin, manganese superoxide dismutase enzyme, and anti-apoptotic Bcl-2 protein (Wajant et al., 2003; Kamata et al., 2005). This cytokine can stimulate microglia glutaminase to release glutamate, thus generating excitotoxicity (Takeuchi et al., 2006) and promoting the development of neurodegenerative diseases. In AD, the direct role of this cytokine remains uncertain; however, TNF-α could be associated with an increased β- and γ –secretase enzyme expression (Blasko et al., 2000; Liao et al., 2004). *In vitro* models have shown that TNF-α directly stimulates BACE1 expression, thus favoring APP processing (Yamamoto et al., 2007). The use of soluble TNF-α inhibitors had a protective effect on APP deregulation by decreasing amyloid aggregate formation and attenuating the cognitive impairment observed in 3xTgAD mice exposed to chronic peripheral inflammation (McAlpine et al., 2009). However, a long-term suppression of the TNF-α receptor signaling pathway can suppress the microglial ability to efficiently remove Aβ aggregates, thus favoring its aggregation at early stages (Montgomery et al., 2011).

### TGF-β

TGF-β; is another cytokine with pleiotropic functions, which has an anti-apoptotic function and promotes neuronal survival. It is believed that this cytokine is involved in neurodegenerative diseases (Krieglstein et al., 2002; Konig et al., 2005). It has been reported an increase in TGF-β 1 levels in the brain of AD patients (Flanders et al., 1995). Animal models (hAPP/TGF-β 1) have shown that TGF-β 1 overexpression promotes amyloidogenesis in the meninges and cerebral vasculature at early stages (2–3 months) (Wyss-Coray et al., 1997). By contrast, in hAPP/TGF-β 1 transgenic mice parenchyma, there is a decrease in amyloid plaque formation, which correlates with microglial activation (Wyss-Coray et al., 2001). In AD patients, although TGF levels are elevated, there is a significant decrease in the expression of the TGF-β receptor type II (Tβ RII); this decrease may suggest that the signaling pathway mediated by this receptor has significant neuroprotective functions and could be altered during the development of the disease (Tesseur et al., 2006).

In summary, the role played by cytokines remains controversial. On one hand, cytokines might favor microglial and astrocytic activation to promote Aβ phagocytosis and neuronal survival. On the other hand, chronic cytokine production has been described as contributors of neurodegeneration.

### Chemokines

Chemokines are small proteins whose principal function is to attract monocytes, macrophages, lymphocytes, neutrophils, basophils, eosinophils, and dendritic cells toward sites in which an inflammatory response is required. Chemokines function through the activation of their G protein-coupled receptors and are divided into four families, CXC, CC, C, and CX_3_C (Wells et al., 1998; Cyster, 1999).

Astrocytes and microglial cells, are the main producer of chemokines in the CNS, and their receptors are observed to be present in neurons. Chemokines and their receptors also participate in the CNS immune response and promote lymphocyte migration from the lymphoid organs in order to establish the inflammatory process (Ransohoff et al., 1996).

Evidence of the participation of chemokines in AD is the presence of monocyte chemotactic proteins (MCP-1 or CCL2) and chemokine receptors CCR3 and CCR5 in reactive microglia that surround senile plaques of AD patients. By contrast, the macrophage inflammatory protein 1α (MIP-1α) is located mainly in neurons, and MIP-1β is located primarily in astrocytes that surround the plaques (Ishizuka et al., 1997; Xia et al., 1998). The differential expression of chemokines and their receptors promotes glia-neuron communication to establish a local inflammatory response, which could favor Aβ phagocytosis in early stages of AD. Similarly, it is known that this inflammation contributes to Tau pathology and thereby accelerates the disease progression (Zilka et al., 2012).

Chronic inflammation in AD promotes the increased chemotaxis of phagocytic cells, thus favoring microglial recruitment around Aβ aggregates (Yamamoto et al., 2005). A recent study of CSF from AD patients revealed increased CCL2 levels, which correlated with cognitive decline (Westin et al., 2012). Moreover, because of Aβ aggregation, an increase in IL-8 production (CXCL8) is generated in neurons, which correlates with an increase in the formation of brain-derived neurotrophic factor (BDNF) (Ashutosh et al., 2011).

The presence of Aβ, in the microvasculature, promotes the release of IL-8, MCP-1, MIP-1 MIP-1α, and MIP-1β chemokines, and thereby promotes monocyte differentiation into macrophages and their migration through the blood-brain barrier (Fiala et al., 1998). CSF analysis from AD patients also showed an increase in MCP-1 and IL-8 chemokines levels (Galimberti et al., 2003; Correa et al., 2011). Similarly, *in vitro* models have demonstrated the migration ability of lymphocytes (CD4^+^ and CD8^+^) through the blood-brain barrier (Man et al., 2007) because of an increased MIP-1α level.

In conclusion, chemokines in the CNS are able to promote local and peripheral immune system cell migration in order to establish an immune response. In AD, it has been proposed that the chronic production of chemokines contributes to disease progression.

### Cyclooxygenases

Cyclooxygenases (COX) are enzymes responsible for converting arachidonic acid to H2 prostaglandin, which is a PG precursor. In general, these lipidic molecules are involved in inflammation because they cause vasodilation, thus allowing the immune system cell transport to operate at the target site (Williams, 1978). COX-1 and COX-2 isoforms are expressed in mammalian brain. COX-1 is expressed in microglia and neurons located in the pons and spinal cord that perform autonomous and sensory functions. COX-2 occurs in glutamatergic neurons of hippocampus and cortex and is thought to be involved in the modulation of plasticity processes and long-term potentiation (Yamagata et al., 1993; Breder et al., 1995); in pathological conditions, the presence of COX-1 in the CNS has been linked to inflammation development, whereas COX-2 is associated with neurotoxicity. Microglia surrounding the NPs in AD patient shows a high expression of COX-1, which suggests inflammation (Yermakova et al., 1999). On the other hand, COX-2 expression in the hippocampal CA3 region appears to be related with the quantity of NPs and NFTs and the observed cognitive impairment (Ho et al., 2001). COX-2 overexpression in a triple transgenic model (hAPP/PS1/hCOX-2) has resulted in an increase in active Caspase-3 immuoreactivity and retinoblastoma protein phosphorylation (S^795^-pRb). The Rb protein regulates the G1 phase of the cell cycle, but S^725^ phosphorylation suppresses cell growth. *In vitro* studies revealed that hCOX-2 overexpression in primary cultures of cortical and hippocampal neurons derived from transgenic mice, accelerates the apoptotic damage induced by Aβ, causing cell cycle abnormalities (Xiang et al., 2002a). COX-2 also promotes amyloid plaque formation in parenchyma and increases prostaglandin E2 production. This increased plaque formation, correlates with an increase in amyloid peptide formation (Aβ 1–40 and Aβ 1–42) through the γ –secretase activation without affecting the APP expression levels (Xiang et al., 2002b). Studies performed in a mouse model using COX-2^−/−^ deficient mice demonstrated that the inflammatory response mediated by Aβ was diminished, which suggested that COX-2 inhibition could be an important target to be use for therapy (Choi and Bosetti, 2009).

The analysis of post-mortem brain tissue from AD patients demonstrated that the maximum immunoreactivity for COX-2 and ppRb occurred in the early stages of the disease (Braak), before occur the maximum astrocytic and microglial activation. Contrary to *in vitro* observations, the post-mortem tissue analysis did not support the direct relationship between the microglial and astrocytic activation with the neuronal COX-2 and ppRb expression in AD (Hoozemans et al., 2005).

Currently, the cause of alterations in COX-2 levels during different stages of AD, remains unknown. However, it has been suggested that inflammation in the early stages of AD could promote such alterations, and IL-1β could promote an increase in COX-2 expression and PGs production (Hoozemans et al., 2001).

### Nitric oxide

NO is a molecule that contributes importantly in cell signaling. In the presence of oxygen, L-arginine, is converted to L-citrulline and release NO. Nitric oxide synthase (NOS) is the enzyme responsible for catalyzing this reaction, and it occurs in three isoforms. In the CNS, the neuronal isoform (nNOS) is widely distributed in neurons, astrocytes and blood vessels. The endothelial isoform (eNOS) is located in the hippocampal pyramidal neurons, endothelial cells and some astrocytes. The expression of the inducible isoform (iNOS) is typically low but is increased in microglia and astrocytes during neuro-inflammation. Under physiological conditions, it is believed that NO regulates the release of neurotransmitters and hormones and promotes cell survival and long-term potentiation. However, high levels of NO are generated in inflammatory conditions, which might contribute to synaptic transmission dysfunction, protein and lipid oxidative damage, excitotoxicity, and neuronal death (Liu et al., 2002; Bishop and Anderson, 2005; Calabrese et al., 2007).

Tissue and neuronal analyses of AD patients have revealed that Aβ is able to promote NOS expression and NO production in microglia cells and reactive astrocytes (Goodwin et al., 1997; Wallace et al., 1997; Akama et al., 1998). Aβ also promotes the pro-inflammatory cytokines IL-1β and TNF-α liberation which contribute to NO and peroxynitrite formation (Rossi and Bianchini, 1996; Combs et al., 2001) and cause protein and lipid modifications, mitochondrial damage, apoptosis and promote Aβ formation, increasing the γ-secretase complex activity (Torreilles et al., 1999; Keil et al., 2004; Guix et al., 2012). Under normal conditions, NO is synthesized in the microvasculature and regulates β-secretase activity which participate in APP processing (Austin et al., 2010). NOS2 also favors Aβ elimination by regulating MMP-9/TIMP-1 expression, which is a key enzyme that degrades amyloid (Ridnour et al., 2012). In addition, it has been shown that chronic NO formation may alter insulin-degrading enzyme activity (Kummer et al., 2012). NO synthesis during AD development could also contribute to NFT formation. In co-cultures of astrocytes and hippocampal neurons of rats, exposure to Aβ 25–35 was found to increase NO levels, which correlated with increased hyperphosphorylated Tau protein. Interestingly, the use of a NOS inhibitors reduced Tau phosphorylation levels (Saez et al., 2004).

## Inflammation modulator treatments: therapeutics in AD

In the early 1990s, studies began to emerge relating the inflammation process with AD. Studies such as those of Fillit et al. (1991) showed that certain pro-inflammatory cytokines were elevated in AD patients.

Epidemiological studies (Li et al., 1992) indicates that subjects with arthritis have a lower prevalence of AD, after which it was proposed that this negative association could be related to the chronic use of anti-inflammatory drugs (Dickson and Rogers, 1992). From these and other results, these drugs began to be proposed as a new therapeutic approach for AD treatment. Currently, numerous epidemiological studies, experimental *in vitro* and *in vivo* models and clinical trials have been designed to elucidate the relationship between modulators of inflammation and AD.

Typically, anti-inflammatory agents are divided into non-steroidal anti-inflammatory drugs (NSAIDs) and glucocorticoids. This section will review the mechanisms of action of both types of anti-inflammatory agents and their role in AD and the results of some of the most relevant epidemiological and experimental studies. This section will also analyse the use of active and passive immunotherapy as a therapeutic strategy for AD (Table 1).

**Table 1:**
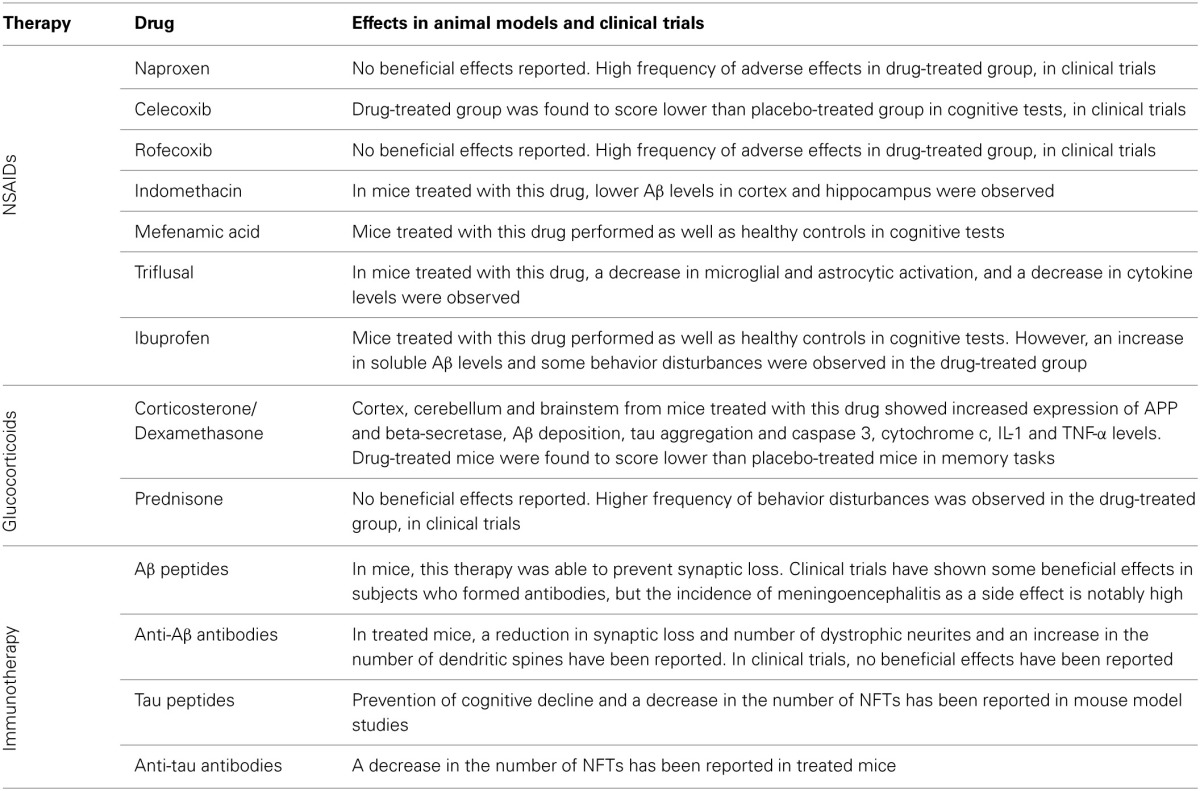
**Inflammation modulator treatments and their effects in animal models and clinical trials**.

Although most of these treatments have been successful in animal models, these beneficial results have not been reproduced in clinical trials, and even have been cause of detrimental effects.

### Anti-inflammatory drugs

NSAIDs are a heterogeneous group of drugs that share a common mechanism of action, which involves COX-1 and COX-2 enzymes inhibition either selectively or non-selectively (Vane and Botting, 1987). These enzymes catalyze arachidonic acid conversion to prostaglandin H_2_ (PGH_2_), which is subsequently converted into various PGs (PGE_2_, PGD_2_, PGF_2__α_, and PGI_2_) and thromboxane (TX) (Dubois et al., 1998).

PG and TX synthesis increases in occurrence of tissue damage, acting as inflammation mediators, increasing blood flow and vasodilation in the damaged tissue and increasing microvascular permeability (Flower et al., 1976).

The results of several epidemiological studies designed to identify AD risk factors have shown that prolonged use of NSAID could reduce AD risk. The following are the results from some of the most representative studies conducted on different worldwide populations.

The *Sydney Old Persons Study* showed that NSAID use, is significantly lower in subjects who developed AD than in those who did not develop any type of dementia during the study period (Broe et al., 2000). Notably, NSAID use is not associated with any other type of dementia except AD. This result could suggest that NSAID use in AD may act through a different mechanism from their properties as cyclooxygenase inhibitors. The Rotterdam study (In'T Veld et al., 2001), Multi-Institutional Research Alzheimer's Genetic Epidemiology Study (Yip et al., 2005) and Canadian Study of Health and Aging (CSHA) (Coté et al., 2012) also observed an association between the NSAID use and a reduced AD risk. Szekely et al. conducted a meta-analysis of six studies (the Baltimore Longitudinal Study of Aging, Cache County Study, CSHA, Cardiovascular Health Study, Framingham Heart Study and Monongahela Valley Independent Elders Study) and reiterated the association between the NSAID use and a reduced AD risk (Szekely et al., 2008). However, some studies have failed to replicate this association. The Longitudinal Aging Study Amsterdam observed a reduction in AD risk associated with the use of aspirin alone and observed no association between AD risk and other NSAIDs (Jonker et al., 2003). Bendlin et al. also observed no significant differences in the results of neuropsychological memory and learning tests between NSAIDs users and non-users (Bendlin et al., 2010).

Other studies have observed controversial results, such as Fourrier et al. who observed an association between NSAIDs use and a decrease in Mini-Mental State Examination (MMSE) scores (Fourrier et al., 1996). Similarly, Breitner et al. reported a greater incidence of AD among NSAIDs users compared with controls (Breitner et al., 2009).

Martin et al. obtained equally unfavorable results when testing the effect of naproxen and celecoxib in older adults with a family history of AD (Martin et al., 2008). In this study, subjects treated with naproxen and those treated with celecoxib scored lower on the MMSE than subjects treated with placebo, which suggested a detrimental effect of the use of these drugs on cognitive performance. Aisen et al. tested the effect of rofecoxib and naproxen on patients with mild to moderate AD (Aisen et al., 2003). The study, which lasted one year, showed no significant differences in the performance of subjects treated with rofecoxib or naproxen or placebo. In addition, subjects in NSAID-treated groups reported a high frequency of adverse effects such as fatigue and dizziness and had a significantly higher incidence of hypertension. Another study with rofecoxib, conducted in patients with mild cognitive impairment (MCI), suggested a deleterious effect of rofecoxib (Thal et al., 2005).

The results of AD animal model studies appear to support the hypothesis that the use of NSAID is beneficial, not only as a method of preventing the disease, but also as a therapeutic strategy. This support was observed in Tg2576 (Swe-APP) transgenic mice treated with indomethacin, which showed a marked decrease in Aβ levels (Aβ_1−40_ and Aβ_1−42_) in both cortex and hippocampus (Sung et al., 2004).

Subsequently, Joo et al. showed that mice treated with mefenamic acid for 3 weeks after being treated with Aβ 1–42 or expressing Swe-APP, re-established their performance in the Morris water maze test in a comparable way to the vehicle-treated group (Joo et al., 2006). Coma et al. obtained similar results in Tg2576 transgenic mice treated with Triflusal (a NSAID from the salicylate family but a non-aspirin derivative), thus re-establishing transgenic mice (Tg+) performance in Morris test and a conditioning test (Coma et al., 2010). Although Triflusal had no effect on reducing NP size (NPS) or number, it reduced the amount of activated astrocytes and microglia and IL-1β and TNF-α levels in the hippocampal CA1 region and entorhinal cortex. Van Dam et al. observed similar results in TgAPP23 mice after treatment with Ibuprofen (Van Dam et al., 2010).

In the animal model (5XFAD), which overexpressed the Swedish double mutation (K670N, M671L), Florida mutation (I716V), London mutation (V717I), and double mutation in PS1 (M146L and L286V), treatment with ibuprofen for 3 months resulted in a reduction of the inflammatory response. However, it is notable that there was an increase in soluble Aβ 42 levels and there was some changes in behavior, thus questioning whether anti-inflammatory drug use may actually be beneficial for AD treatment (Hillmann et al., 2012).

Whereas inflammation is a neuropathological feature always present in AD, and inflammatory processes significantly contribute to cell damage in this disease, previous studies performed *in vivo* and *in vitro* suggest that NSAIDs participation in AD occurs not only through an anti-inflammatory mechanism but also by modulating Aβ synthesis and removal in the CNS. Several studies have shown that NSAIDs stimulate the non-amyloidogenic APP-processing pathway (Avramovich et al., 2002), decrease β-secretase levels (Sastre et al., 2006), and reduce the Aβ formation (Hirohata et al., 2005).

Although animal models appear to show that NSAIDs are a promising therapeutic option, currently, no clinical trials conducted with these drugs have achieved favorable results in AD patients.

### Glucocorticoids

Similar to NSAIDs, glucocorticoids have potent anti-inflammatory effects, which have made them strong candidates for AD treatment. Basically, the mechanism of action of glucocorticoids is based on their ability to bind to its receptors (Glucocoticoid Receptor, GR), which is observed in the cytoplasm in its free form, which translocates into the nucleus after binding to one of its ligands. When GR locate at the nucleus, it binds to certain nucleotide sequences, glucocorticoid response elements (GREs), which are located in the promoter region of a variety of genes.

Depending on the type of GRE sequence to which it binds, GR will positively or negatively regulate gene expression (Beato et al., 1989). The importance of this binding in the inflammatory process is that some genes which GR binds to and activates, have anti-inflammatory effects such as annexin-1 (lipocortin-1), interleukin 10 and NF-κ B inhibitor (Iκ B-α) (Barnes, 2006). In addition, it has also been suggested that glucocorticoids interact directly with Aβ.

The study of post-mortem AD brains showed a decrease in the presence of NPs in subjects under chronic treatment with corticosteroids. No significant differences were observed in the number of NFTs. This study also reported no association between chronic treatment with NSAIDs and the number of NPs (Beeri et al., 2012).

Despite its anti-inflammatory effect, the use of glucocorticoid as a therapeutic strategy in the treatment of AD is controversial, because numerous studies have associated increased levels of glucocorticoids (cortisol), to an increased AD risk. In 1994, Swaab et al. measured cortisol levels in post-mortem samples of CSF and observed an 83% increase in AD subjects compared to healthy controls (Swaab et al., 1994). These results coincide with the results of Laske who observed significantly higher levels of serum cortisol in AD patients compared with age-matched healthy controls (Laske et al., 2009).

The majority of animal model experiments does not support the use of corticosteroids as a therapeutic strategy for AD and suggests that these drugs promote the development of the neuropathological features of this disease.

The treatment of rats with glucocorticoids (corticosterone and dexamethasone) induces an increase in APP expression in cortex, cerebellum, and brain stem (Budas et al., 1999), thus suggesting an adverse effect. Additionally, there is an increase in Aβ formation because of the increased APP and β-secretase levels. These glucocorticoid levels also correlate with Tau accumulation (Green et al., 2006), memory, and learning impairment (Yao et al., 2007) and increased levels of caspase 3 and cytochrome c, which indicates the presence of a pro-apoptotic enviroment (Li et al., 2010). Finally, high levels of corticosterone in hippocampal cells have a pro-inflammatory effect, thus favoring IL-1 and TNF-α expression (Macpherson et al., 2005).

These studies indicates that the mechanisms that might associate glucocorticoids with an increase in AD risk are related with the increasing expression of proteins involved in Aβ synthesis (APP and β-secretase) or promoting apoptosis (Cotman and Anderson, 1995).

In humans, there is only one clinical trial investigating the effect of glucocorticoids in the treatment for AD, the trial investigate the used of a daily dose of prednisone or placebo for 1 year (Aisen et al., 2000). There were no significant differences in the cognitive performance of AD subjects treated with prednisone or placebo at the end of treatment; however, the prednisone-treated subjects showed a higher frequency of behavioral disturbances compared to controls.

In general, there is little evidence to suggest the neuroprotective or therapeutic role of glucocorticoids in AD; in contrast, there is extensive evidence of a possible neurotoxic role. Whereas it is possible that under certain conditions these substances are actually neuroprotective, details of their mechanism of action should be extensively evaluated in AD before a clinical testing protocol can be considered.

### Immunotherapy

Currently, there is no consensus among researchers regarding what triggers AD pathophysiology or which events are causes and which are bodily responses to counteract the damage caused by the disease. This problem has also been raised regarding inflammation.

Whereas therapies using NSAIDs and glucocorticoids were designed based on the idea that the immune response in AD has an adverse effect (Blasko et al., 2004) or may be a causal factor (McGeer and McGeer, 1995), there are other approaches which suggest that the immune response may be beneficial because it attempts to counteract the deleterious effects of Aβ oligomers and Tau aggregation.

Immunotherapy is one of the therapeutic strategies designed under these bases because it is underlying on promoting the immune response against the altered molecules that are present in the most distinctive histopathological lesions of the disease (NPs and NFTs) and destroy them. This section will review the types (anti-Aβ and anti-Tau immunotherapy) and some results of immunotherapeutic studies with AD patients.

### Anti-Aβ immunotherapy

Animal studies in which the anti-Aβ immunotherapy strategy has been tested have been promising. Buttini observed that either active (using four different Aβ sequence fragments) or passive immunotherapy (using 3D6 anti-Aβ and 12B4 antibodies) prevent the loss of synapses in PDAPP transgenic mice (Buttini et al., 2005).

The application of a single 3D6 anti-Aβ dose in Tg2576 mice demonstrated that there was no reduction in the number of NPs; nevertheless, there was a reduction in the number of dystrophic neurites (Rozkalne et al., 2009). On the other hand, the application of this antibody in PDAPP mice showed an increase in the number of dendritic spines, which suggests that this anti-Aβ immunotherapy approach, can increase neuronal plasticity and could contribute to the recovery of neural circuits. Subsequently, the effect of passive immunotherapy was studied with 7B6 anti-Aβ antibodies in Aβ APPswe/PS1dE9 mice (Spires-Jones et al., 2009; Liu et al., 2011). The authors observed that mice had a higher density of mono-aminergic axons in both the cortex and hippocampus. Immunotherapy using anti-Aβ CAD106 antibodies in different transgenic mouse models for APP (APP23 and APP24) showed a reduction in the presence of Aβ in both models (Wiessner et al., 2011).

In humans, some results suggest that anti-Aβ active immunotherapy could be beneficial; however, the frequency of adverse effects has been too high. In a study of active immunotherapy using Aβ_1−42_ pre-aggregating (AN1792) in combination with immunogenic adjuvant QS-21 (a saponin of vegetable origin) observed a slower cognitive decline in subjects responding to the treatment (forming specific antibodies) (Hock et al., 2003; Orgogozo et al., 2003). However, 10% of the participants developed cases of aseptic meningoencephalitis. A similar result was obtained in an additional group of patients who were evaluated after stopping the treatment due to the adverse effects observed. The monitoring of these patients included cognitive testing and MRI scans to determine brain volume. Patients who were classified as antibody producers before the suspension of the study were tested for the presence of antibodies using ELISA; 89% of patients had antibodies at the time of investigation. These patients positive to antibodies production, performed better on cognitive tests than those treated with placebo, which suggests a possible beneficial effect of the treatment. However, brain volume loss was identical for both groups (Vellas et al., 2009).

The high incidence of aseptic meningoencephalitis among the subjects receiving active anti-Aβ immunotherapy could be attributed to an excessive pro-inflammatory response by T lymphocytes. Unfortunately, passive immunization with bapineuzumab, (a humanized murine monoclonal antibody), showed no differences in cognitive performance in subjects treated with bapineuzumab or placebo (Salloway et al., 2009).

### Anti-Tau immunotherapy

Most of the research efforts into AD immunotherapy have focused on anti-Aβ immunotherapy. However, some studies in animal models have also used anti-Tau immunization.

Boutajangout et al. showed that immunotherapy with the complete 441 amino acids peptides of the human Tau protein (hTau) prevented cognitive decline in an hTau transgenic mouse model, which expressed the hTau transgene (Boutajangout et al., 2010). Boimel et al. immunized the Tau transgenic mice presenting NFTs with phosphorylated Tau peptides. A 40% reduction in the number of NFTs was observed (Boimel et al., 2010). Chai et al. studied the effect of passive immunotherapy for Tau in JNPL3 and P301S transgenic mice. Using PHF1 and MC1 antibodies, the authors observed that the peripheral administration of both antibodies significantly reduced Tau pathology compared to controls (Chai et al., 2011). Although these results suggest that anti-Tau immunotherapy could provide favorable results, no clinical trial has been reported to date that evaluated the real effects on humans.

These results indicate that immunotherapy, particularly anti-Aβ active immunotherapy, could be advantageous when a strategy that minimizes its adverse effects is achieved. Further studies are also required with anti-Tau immunotherapy to evaluate whether this therapy could be functional in the future.

## Conclusion

Currently, several genetic and epidemiological studies have provided an overview of the inflammatory mechanisms involved in AD. Although the molecular basis of the disease remains unknown, the inflammation induced by Aβ has an important role in the neurodegenerative process. The inflammatory process itself is driven by microglial and astrocytic activation through the induction of pro-inflammatory molecules and related signaling pathways, thus leading to synaptic damage, neuronal loss, and the activation of other inflammatory participants.

Although, the role of amyloid as a potential initiator of inflammation is not obvious, its accumulation exerts an indirect effect by activating caspases and transcription factors, such as NF-κ B and AP-1, which produce numerous inflammation amplifiers (IL-1β, TNF-α, and IL-6). Pro-inflammatory cytokines, such as TNF-α and IL-1β and IL-6, could act directly on the neuron and induce apoptosis. Similarly, TNF-α and IL-1β can activate astrocytes, which could release factors that have the capacity to activate microglia.

Furthermore, APP, BACE1, and PSEN expression is governed by factors such as NF-κ B. The genes encoding these proteins have sites in their promoter regions, which are recognized by NF-κ B; in turn, the expression of these factors is upregulated by the presence of pro-inflammatory cytokines.

Inflammatory mediators acting on neurons contribute to an increase in amyloid production and activate microglia-mediated inflammation. The microglia-neuron communication amplifies the production of factors that contribute to AD-type pathology. However, the neural response is specific for the receptor type expressed in the different neuronal populations. For example, TNF-α binds TNFR1, which activates the cell survival pathway through NF-κ B and the apoptotic pathway through the activation of caspases. Conversely, TNFRII signaling only activates NF-κ B.

This cascade is primarily mediated by the pro-inflammatory cytokine IL-1β, which is expressed by microglia cells. IL-1β may cause neuronal death via various pathways, which activate microglia and consequently increase the release of IL-1β, thus generating a self-sustaining mechanism that is amplified by itself. This slow but steady inflammation state, generated for long periods in the brain eventually can destroy neurons and contribute to the clinical symptoms observed in the disease (Figure 1).

Finally, in accordance with all the above data, particularly because the results of the treatments used have been contradictory so far and there are no clinical trials that shown that anti-inflammatory treatments and the use of immunotherapy are completely safe or beneficial, it is necessary to develop and implement new strategies for AD immunological treatments.

### Conflict of interest statement

The authors declare that the research was conducted in the absence of any commercial or financial relationships that could be construed as a potential conflict of interest.
